# Transcriptional Response and Plant Growth Promoting Activity of Pseudomonas fluorescens DR397 under Drought Stress Conditions

**DOI:** 10.1128/spectrum.00979-22

**Published:** 2022-07-12

**Authors:** Susmita Das Nishu, Jee Hyun No, Tae Kwon Lee

**Affiliations:** a Department of Environmental Engineering, Yonsei University, Wonju, Republic of Korea; USDA - San Joaquin Valley Agricultural Sciences Center

**Keywords:** drought stress, plant growth promotion, *Pseudomonas*, whole-genome sequence, RNA-seq, legume, compatible solutes

## Abstract

Drought is one of the most vulnerable factors that affect crop productivity. Little is known about plant-associated microbiomes and their functional roles in assisting plant growth under drought. We investigated the genetic and transcriptomic characteristics of opportunistic beneficial microorganisms that selectively alleviate stress through plant-bacteria interactions under drought. Pseudomonas fluorescens DR397 was isolated from the drought-prone rhizospheric soil of soybean and showed high metabolic activity at −1.25 Mpa. The genome of DR397 possesses several genes related to the synthesis of compatible solutes (choline and glycine-betaine), exopolysaccharides (alginate and cellulose), and secretion systems (type II, III, IV, and VI), as well as genes related to plant growth promotion (indole-3-acetic acid, transketolase, and thiamine phosphate synthesis). The expression of these genes was significantly upregulated (8- to 263-fold change) only under drought conditions with plant root exudate treatment, whereas subtle transcriptomic changes were observed under solely root exudate treatment. When DR397 was placed on both legume cultivars (Pisum sativum and Phaseolus vulgaris), growth was hardly affected under well-watered conditions, but the shoot and root growths were increased by up from 62.0% to 149.1% compared with the control group under drought conditions. These results provide fundamental insight on the plant-bacterial interactions that alleviate plant stress as an important ecological strategy for improving drought tolerance.

**IMPORTANCE** Drought is a serious abiotic stress on plants as wells as the microbes that coexist with plants, which significantly lowers their fitness. The plant-bacterial interaction is an important strategy to enhance their fitness under drought. However, many knowledge gaps still exist in our understanding of transcriptomic features of bacteria interacting with plant under drought. Here, by investigating the transcriptomic profiles and pot cultivation with legume, we show that the interactions of Pseudomonas fluorescens DR397 with plants change with drought. We, therefore, provide a fundamental evidence of a hidden hero in the soil that promote plant fitness from external stress.

## INTRODUCTION

Drought, an extreme meteorological phenomenon, is the main climatic limitation to global agricultural productivity (1). A single drought event can reduce agricultural gross domestic output by up to 0.8% on average globally, with varying magnitudes of damage per country (2). A total of 454 million hectares of land, approximately three-fourths of the global harvested area, experienced yield losses due to drought between 1983 and 2009, resulting in a cumulative yield loss of approximately $166 billion (2). Drought reduced wheat and maize yields by 21% and 40%, respectively, on a global scale from 1980 to 2015 (3). In 2018 alone, drought cost China 48.362 billion yuan in direct economic losses, 15.697 billion yuan in grain losses, and 8.466 billion yuan in cash crop losses (4). To avoid catastrophic consequences for global food security, a stronger emphasis on building crop drought resilience and adaptive capacity to cope with future droughts is required. Legitimate investigation of biotechnological approaches and their accurate implementation into drought adaptation features in crops are urgently needed to address severe drought challenges in agriculture.

Inoculation of drought-tolerant, plant growth-promoting rhizobacteria (PGPR) is a promising method over other conventional methods in conferring beneficial effects to plants (5). PGPR are known to promote plant growth and alleviate stress (6). Pseudomonas spp. has been extensively studied and has been shown to reduce abiotic stresses in plants (7, 8). In particular, the rapid utilization of root exudates by Pseudomonas spp. benefits root colonization and plant growth-promoting activity (9). However, the survival ability of inoculated bacteria outcompetes that of the native microflora, and rhizosphere colonization remains a crucial step for a successful application under drought (5). To accurately understand the properties of PGPR, it is necessary to study how they respond genetically to drought situations. Analysis of whole genomes and transcriptomes of drought-tolerant rhizobacteria is a promising approach to identify the underlying molecular mechanisms that regulate plant acclimation to drought and the maintenance of productivity under stressful conditions (8).

Plants have evolved complex morphological and metabolic responses to drought stress, which have a notable impact on root microbial communities. Both plant and microbes alter their metabolisms based on the available carbon pool, which controls other activities such as synthesizing compatible solutes and activating defense pathways (6). Drought causes a shift in the photosynthetic process, resulting in drastic changes in metabolite abundance and carbon output in the soil (10). Microorganisms have a wide range of evolutionary adaptations and physiological acclimation mechanisms that allow them to live and thrive in harsh environments (11). How drought challenges microbial function, triggers them to resource allocation, or regain dormant metabolic activities are rarely known. We anticipated that drought induces stress-tolerant microorganisms to perform more beneficial roles in symbiotic interactions that are normally dormant under well-watered conditions to improve the host plant’s fitness during drought.

In this study, we analyze underlying symbiotic interaction mechanism in P. fluorescens DR397 with legumes exposed to drought stress, using RNASeq to identify key genetic mechanisms at transcriptome level. Strain DR397 was tested for its contribution to plant growth under drought by cultivating with two different legume plant phenotypes (Pisum sativum and Phaseolus vulgaris). Our study emphasizes the molecular mechanism of intricate regulatory gene networks in DR397 to improve drought tolerance in legumes. These findings will aid in a better understanding of drought-induced plant-microbe interactions.

## RESULTS

### Drought tolerance and the plant growth-promoting ability of DR397.

**FIG 1:**
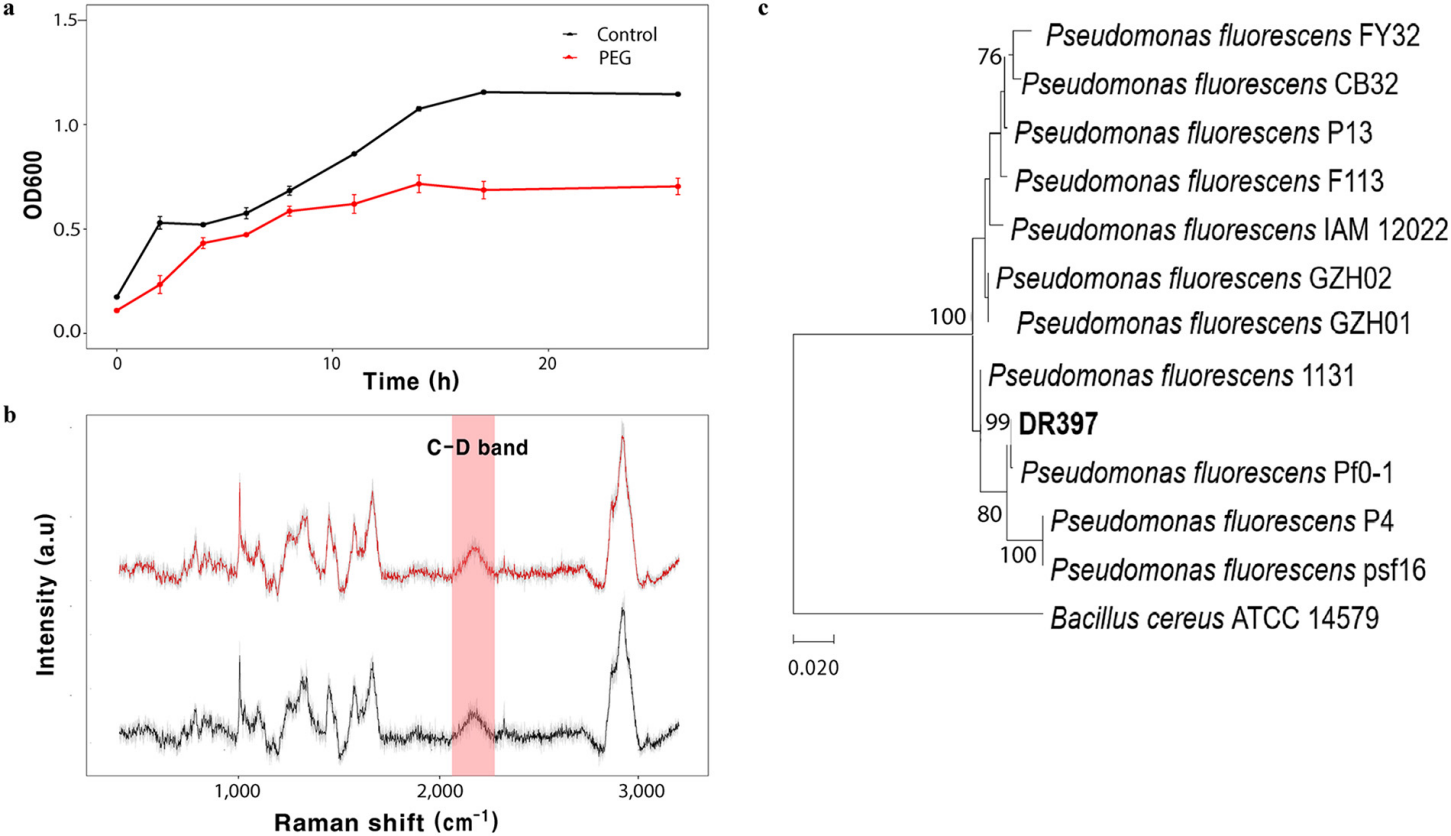
Growth, metabolic activity, and phylogenetic affiliation of strain DR397. (a) Growth curve of strain DR397 with 0% and 25% polyethylene glycol (PEG; PEG6000) (−1.25 Mpa). (b) Single-cell Raman spectra (SCRS) of DR397 after 24 h of incubation in medium with 0% and 25% PEG including 40% deuterium water. The black and red lines represent the control and PEG treatment, respectively. (c) A neighbor-joining method-based phylogenetic tree constructed with 16s rRNA sequence of strain DR397 (bold) and other closely related species, including previously reported plant growth-promoting Pseudomonas species available from the NCBI GenBank database. The scale bar represents expected changes per site.

Strain DR397 showed remarkable growth in the medium treated with 25% polyethylene glycol (PEG), based on OD_600_ (Fig. 1a). After 24 h, the strain was able to grow to approximately 61% of the maximum growth of the control under PEG treatment. Using Raman spectroscopy, DR397 was confirmed to maintain its metabolic activity like those of controls under PEG treatment by detecting the C-D band (2,040 to 2,300 cm^−1^) in the Raman spectra (Fig. 1b). The phylogenetic tree constructed using the neighbor-joining method revealed that strain DR397 clustered with the genus Pseudomonas (Fig. 1c), sharing 99.0% similarity of its 16S rRNA with the soil-dehydrated stress-tolerant rhizobacterium P. fluorescens Pf0-1 (12).

P. fluorescens DR397 produced 26.5 μg/mL of indole-3-acetic acid (IAA) and 403.5 μg/mL of soluble phosphate *in vitro.* The strain was able to secrete siderophores, as determined by chrome azurol sulfonate (CAS) assay. Therefore, this strain exhibit the potential to promote plant growth by generating IAA, siderophores, and solubilizing phosphate (13).

### Genomic features of P. fluorescens DR397.

**FIG 2:**
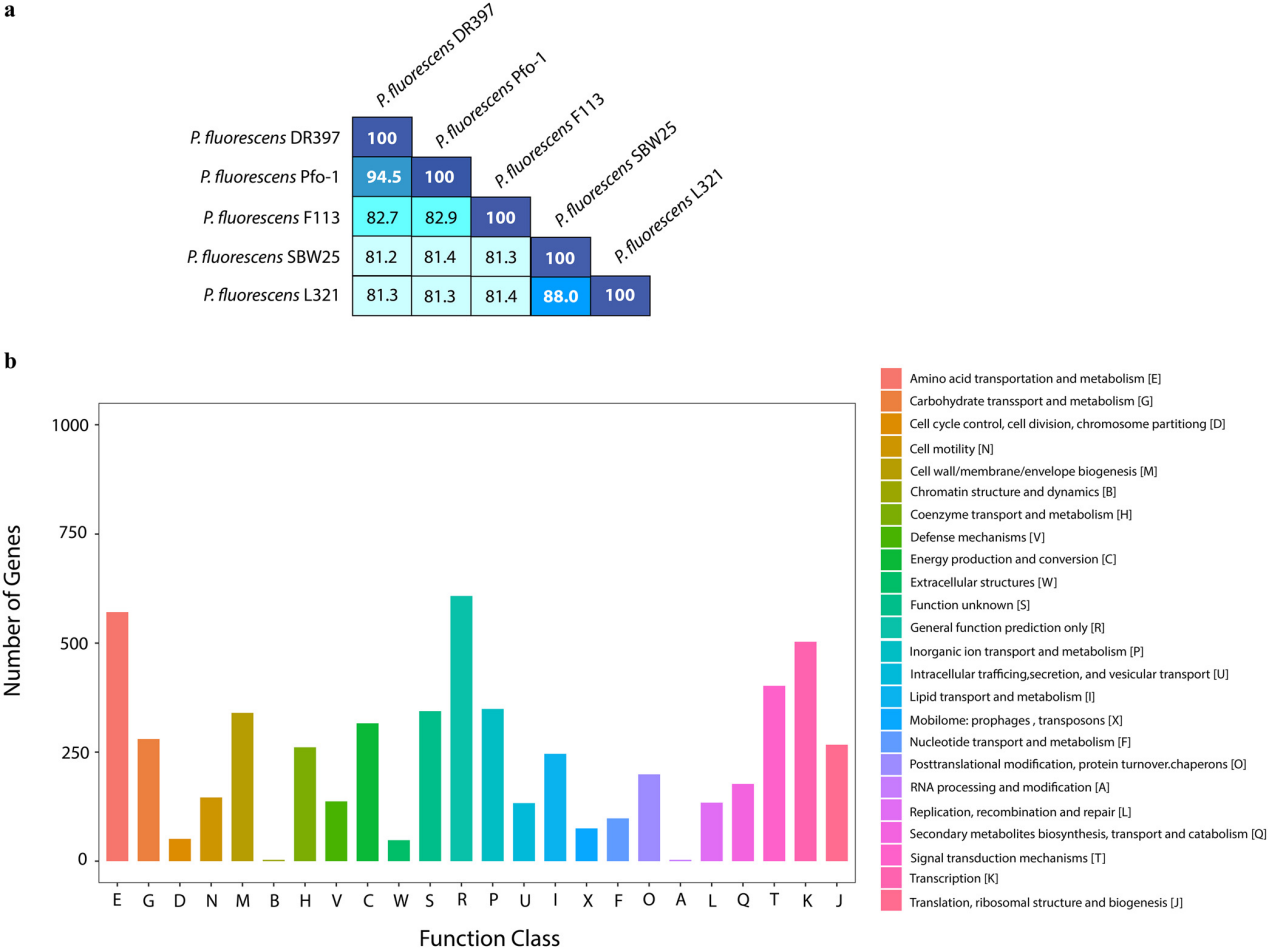
Average nucleotide identity (ANI) and functional classification in clusters of orthologous groups of proteins (COG). (a) ANI between the strain DR397 with other plant growth-promoting Pseudomonas fluorescens strains. (b) COG functional gene distribution in DR397.

The genome of DR397 contains one circular chromosome (6,418,441 bp) with a GC content of 60.6% and is comprised of 5,682 coding sequences. The genome was complete with no detectable contamination (0%). The general genomic features are summarized in Table S1. The average nucleotide identity (ANI) score revealed 81.2% to 94.5% similarity between DR397 and other plant growth-promoting P. fluorescens strains (Fig. 2a). DR397 shared the highest genomic similarity (94.5%) with Pseudomonas sp. *P*f0-1, an isolate which was able to survive under dehydration stress in the rhizosphere (11). Among all the predicted CDSs, 4,715 genes were classified into 24 categories of clusters of orthologous groups (COG) families (14) (Fig. 2b). The DR397 genes were assigned to the following systems: E (amino acid transport and metabolism, 10.0%), K (transcription, 8.8%), T (signal transduction mechanism, 7.0%), and P (inorganic ion transport and metabolism, 6.1%).

**FIG 3:**
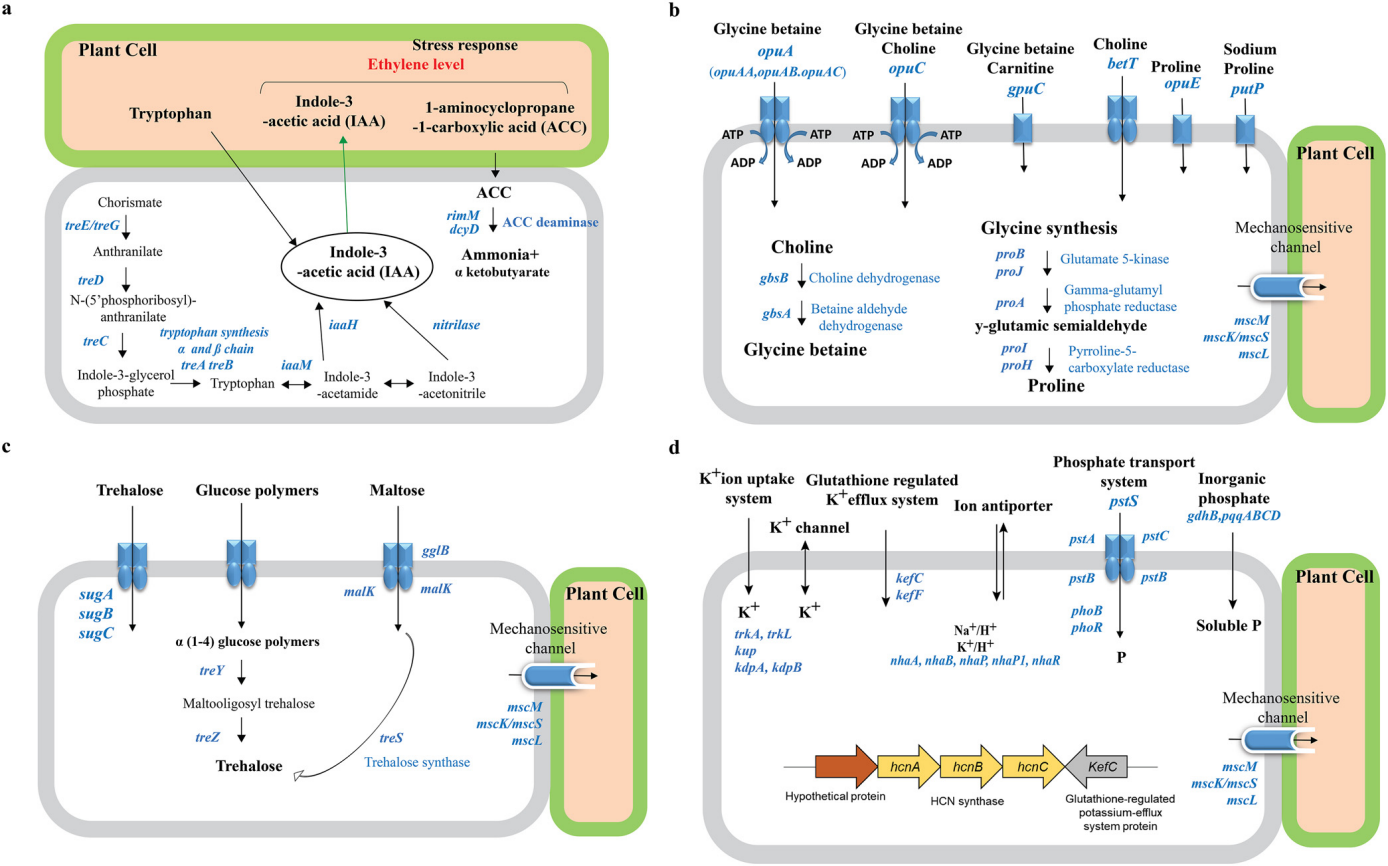
Metabolic pathways and transport systems found in the genome of Pseudomonas *fluorescence* DR397. (a) Gene clusters and functional pathway associated with biosynthesis of indole-3-acetic acid (IAA) and 1-aminocyclopropane-1-carboxylic acid (ACC) deaminase to control plant ethylene levels. (b) Gene clusters for the biosynthesis and uptake of compatible solutes (amino acids): glycine betaine, choline, proline, glutamate in DR397. (c) Compatible solutes (sugar): glucose, maltose uptake, and Trehalose synthesis. (d) Transport and exchange of nutrients, including ions (sodium, potassium) and hydrogen cyanide biosynthesis *hcnABC* structural gene clusters in DR397.

**Genomic insight into adaptive strategies to drought tolerance and plant growth promotion. (i) IAA synthesis and 1-aminocyclopropane-1-carboxylate deaminase production.** The genome of DR397 contains a gene cluster involved in IAA synthesis, consisting of a tryptophan synthase α chain (*trpA*), tryptophan synthase β chain (*trpB*), indole-3-glycerol phosphate synthase (*trpC*), anthranilate phosphoribosyltransferase (*trpD*), tryptophan 2-monooxygenase (*iaaM*), and indoleacetamide hydrolase (*iaaH*) (Fig. 3a). IAA is the main auxin in plants that regulates cell enlargement and division, tissue differentiation, stress signaling, and adaptation (15). The genes of *trpAB* produces tryptophan or enables the generation of indole as an intermediate, which proceeds via *iaaM* expression and catalyzes indole-3-acetamide to IAA using *iaaH* (16). The ribosome maturation factor (*rimM*) and d-cysteine desulfhydrase (*dcyD*) encode 1-aminocyclopropane-1-carboxylate (ACC) deaminase, reducing the ethylene level of plants under stressful conditions (17).

**(ii) Compatible solutes.** We found several genes related to transporters and the biosynthesis of compatible solutes, which could be involved in cellular defense and osmotic balance (18). Gene clusters of osmostress-responsive compatible solutes of both amino acids and sugar were found in DR397 (Fig. 3b and c). DR397 also possessed an ABC-type uptake system, which comprised the substrate-binding glycine betaine protein encoded by o*puA*, glycine betaine choline transporter (*opuC*), glycine betaine/carnitine transporter (*gbu*), osmoregulated proline transporter (*opuE*), high-affinity sodium proline transporter (*putP*), and low-affinity high-capacity osmoregulatory choline transporter (*betT*) (Fig. 3b). Choline dehydrogenase (*gbsB*), betaine dehydrogenase (*gbsA*), and one gene cluster (*proB*, *proA*, and *proH*), for the conversion of choline to glycine betaine, represent the pathway for proline synthesis from glutamate in DR397. This strain possesses genes encoding various mechanosensitive channels that regulate cell osmotic potential by releasing ions and organic compounds with a small conductance (*mscS/mscK*), miniconductances (*mscM*), and a large conductance (*mscL*).

DR397 carries the trehalose transport system permease protein (*sugABC*) and uses two routes (*treY-treZ* and *treS*) for trehalose biosynthesis, which confers cell protection and preserves functionality against multiple stresses (Fig. 3c). In *the treY-treZ* pathway, α-(1, 4)-glucose polymers are converted to trehalose by hydrolyzing trehalose disaccharide, whereas the trehalose synthase/amylase gene (*treS*) isomerizes maltose to trehalose.

**(iii) Transport and exchange of ions.** The DR397 genome comprises various types of ion and nutrient transporters that contribute to the maintenance of ionic hemostasis during stress (Fig. 3d). It carries two copies of trk-type potassium uptake systems (*trkA* and *trkI*), a low-affinity potassium transport system protein (*kup* and *kdp*), and a glutathione-regulated potassium efflux system (*kefC* and *kefF*). We found that inorganic ion membrane transport proteins, including Na(^+^)/H(^+^) antiporters (*nhaA* and *nhaB*) and K(^+^)/H(^+^) antiporters (*nhaP* and *nhaP1*), play key roles in monovalent inorganic cation homeostasis. The phosphate-specific transport system *pst* operon is composed of a binding protein (*pstS*), two integral inner membrane proteins (*pstC* and *pstA*), and ATP-binding proteins (*pstB1* and *pstB2*). The genome also carries glucose dehydrogenase (*gdhB*) and the cofactor pyrrolo-quinolone quinine (*pqqABCD*) gene cluster, which are involved in the solubilization of mineral phosphates fixed in soil particles. We also identified genes (*hcnA*, *hcnB*, and *hcnC*) involved in biosynthesis of hydrogen sulfide (H_2_S), which is essential for plant development signaling and stress responses.

Multiple gene clusters confirmed that P. fluorescens DR397 has the potential to produce a wide array of secondary metabolites and extracellular polymeric substances (EPS) (Table S2). Secondary metabolites, including osmoprotectants, arylpolynenes, and siderophores, are involved in plant growth promotion and induce drought tolerance. The ability to synthesize EPS, such as alginate and cellulose, could have an advantage in out-competing native microbes to adapt well to the plant rhizosphere (9).

### Transcriptomic profiling for differentially expressed genes in response to drought and plant root exudates.

The number of differently expressed genes (DEGs) was much higher when DR397 was treated with PEG regardless of the presence of root exudates (Fig. S1). PEG treatment considerably altered the expression of 1,702 genes (upregulated genes: 785, downregulated genes: 917), whereas the combined treatment of PEG and root exudates reduced the total number of expressed genes, with an increase in upregulated genes (1,009) and a decrease in downregulated genes (641). Under PEG treatment, the number of downregulated genes was higher than that of the upregulated genes whereas the trend was opposite under the combined treatment of PEG and root exudates. Effect of treatment with root exudate on gene expression in DR397 was relatively less (upregulated genes: 79, downregulated genes: 147) than other treatments. Phenotypic data analysis also supports these data. In growth curve (Fig. S2), treatment of root exudates did not show any significant difference with control, while there was a close similarity in growth rate under the treatment of PEG and combined treatment of PEG and root exudates. In Raman spectroscopy, DR397 was confirmed to maintain its metabolic activity as control under all treatment (Fig. S3a). Comparison of the Raman fingerprint section between different treatments revealed that there was minor difference between any treatments and control (*t* test, *P* < 0.01) (Fig. S3b). Raman shifts which had significantly different spectral intensities in treatments and controls are summarized in Table S3 (*t* test, *P* < 0.01).

**FIG 4:**
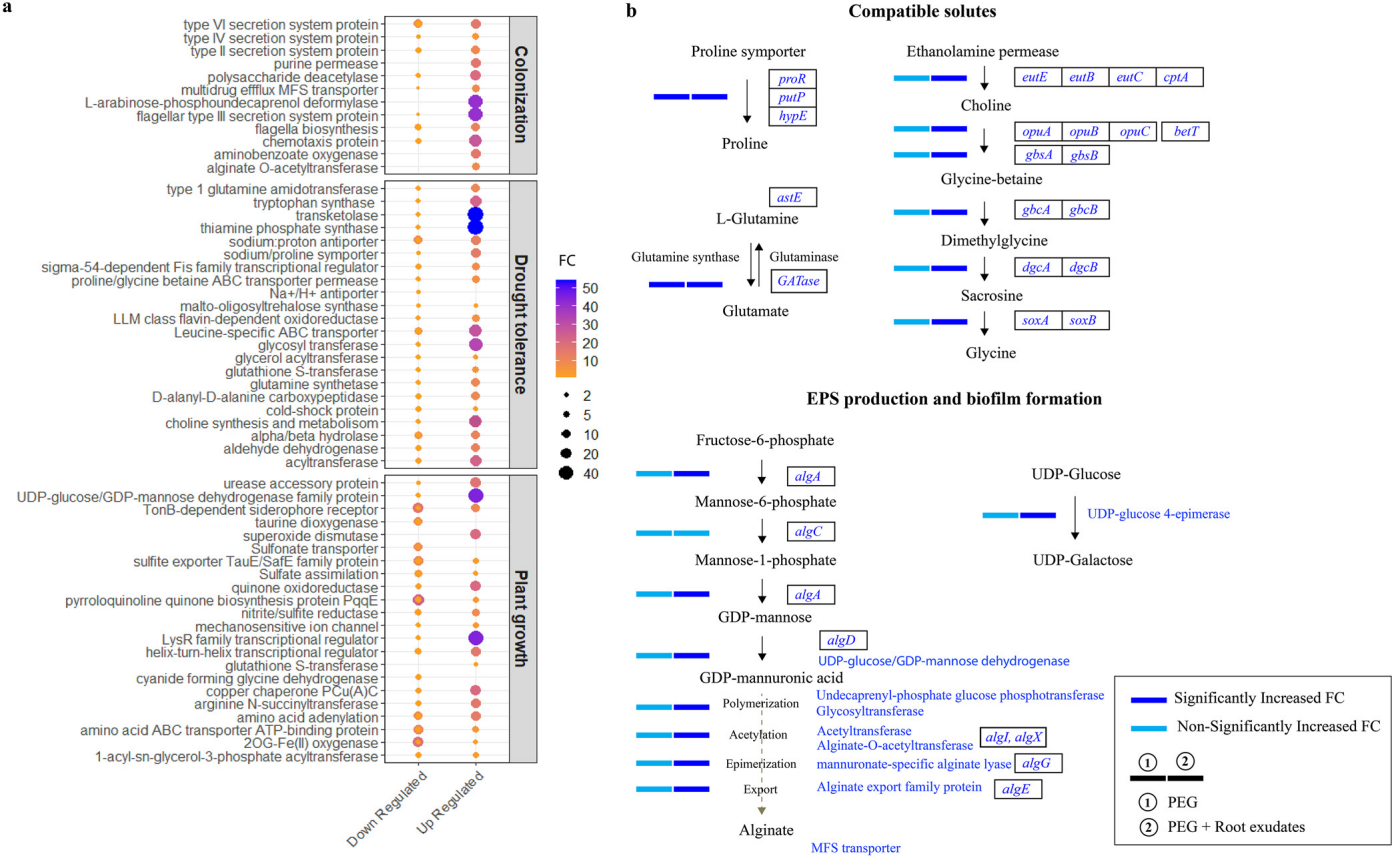
Gene expression of the drought-inducible synergistic functional pathway in DR397. (a) Significantly expressed genes assorted in the functions related to plant root colonization, drought tolerance, and plant growth in DR397 under the treatment of polyethylene glycol (PEG) with root exudates. Both size and colors of the circle representing the FC values. (b) Schematic diagram of drought-induced regulatory pathway through the synthesis and metabolism of compatible solutes and exopolysaccharides (EPS) in DR397 under PEG treatment (first bar) and PEG+Root exudates (second bar). Significant increase, fold change(FC) > 2; nonsignificant increase, FC < 2.

Fifty highly expressed DEGs were selected to compare protein fold changes (FCs) under the three treatments (Fig. S4). The expression of a remarkable number of functional genes was upregulated up to 40-fold under the combined treatment. Fifty selected DEGs were grouped into three categories: root colonization, drought tolerance, and plant growth promotion (Fig. 4a). Treatment with PEG and root exudates increased the expression of genes associated with root colonization for plant-bacteria symbiosis, including secretion systems (types II, III, IV, and VI), purine permease, polysaccharide deacetylase, chemotaxis, arabinose phosphoundecaprenol deformylase, chemotaxis proteins, and aminobenzoate degradation by 20 to 40 FC. Glutamine amidotransferase, tryptophan synthase, transketolase, thiamine phosphate synthesis, leucine-dependent ABC transporter, glyceryl transferase, choline synthesis, and acyltransferase, which are essential for drought tolerance, exhibited markedly upregulated expression, ranging from 7.8 to 54 FC (Fig. 4a). Expression of genes related to multiple compatible solutes such as choline, glycine betaine, glutamine, biosynthesis, and proline transporters was notably upregulated. A schematic diagram was drawn to elaborate on the regulation of gene clusters in the synthesis and metabolism of compatible solutes (Fig. 4b). The fold changes in the expression levels these gene clusters are shown in the supplemental material (Fig. S5).

Expression levels of urease transporters, the UDP-glucose/GDP-mannose dehydrogenase protein family, superoxide dismutase, quinone oxidoreductase, LysR-type transcriptional regulator, and arginine N-succinyltransferase and amino acid adenylation, which correspond to the active participation of strain DR397 in plant growth, increased by up to 45 FC (Fig. 4a). The most remarkable upregulation was noted in the expression of the following genes: UDP-glucose/GDP-mannose dehydrogenase (263.7 FC), UDP-glucose 4-epimerase *galE* (138.1 FC), dihydroxy-acid dehydratase (108.5 FC), undecaprenyl-phosphate glucose phosphotransferase (45.2 FC), alginate O-acetyltransferase (9.5 FC), and 34 genes from the MFS transporter family (FC up to 266.2), which play crucial roles in bacterial biofilm formation for colonization on the root (19, 20). A functional pathway was drawn to understand the regulation of gene clusters in the synthesis of EPS (Fig. 4b). The upregulation of expression of genes involved in the synthesis of EPS, urease, alginate, chemotaxis, flagellar synthesis, and secretion systems is shown in the supplemental materials (Fig. S5, S6). The markedly upregulated expression of genes that play roles in cell adhesion, motility, and biofilm formation indicates the survival strategy of DR397 through symbiotic interactions during exposure to drought stress.

### P. fluorescens promotes the growth of plant phenotypes.

**FIG 5:**
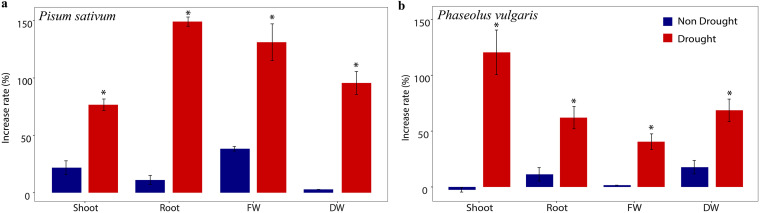
Pseudomonas fluorescence DR397-induced drought tolerance in legume cultivars evaluated by plant cultivation experiment. Effects of P. fluorescence DR397 inoculation on plant growth parameters (shoot length, root length, fresh weight, dry weight) in (a) Pisum sativum and (b) Phaseolus vulgaris. The percentage increase of a growth variable was calculated by comparing the growth with bacterial treatment to the noninoculated control. The blue and red colors indicate plant growth under nondrought and drought settings. Asterisks indicate the level of statistical significance: *, *P* ≤ 0.05.

DR397 improved plant growth regardless of legume cultivar (Fig. 5). Under nondrought conditions, due to inoculation of strain DR397, growth parameters increased to 45% in Pisum sativum while the maximum growth increase in P. vulgaris was up to 20%. DR397 inoculation in Pisum sativum increased shoot length, root length, fresh weight, and dry weight by 76.6%, 149.1%, 131.1%, and 95.6%, respectively, compared with the control under drought conditions. In drought-stressed P. vulgaris, DR397 inoculation increased the shoot length, root length, fresh weight, and dry weight by 120.5%, 62.0%, 40.5%, and 68.6%, respectively. DR397-induced plant growth promotion was significantly higher under drought conditions than under nondrought conditions in both the cultivars.

## DISCUSSION

Consistent drought stress disrupts plant growth, which results in crop yield loss (1). Plants exhibit various adaptive strategies to drought stress by adjusting their metabolism through accumulation of compatible solutes, including choline, proline, glycine-betaines, and trehalose (7). The rhizospheric microbes produce a sizable quantity of compatible solutes that act synergistically with plant-produced compatible solutes for drought tolerance (10). However, there is limited information on how microbes play role in beneficial plant-microbe interactions under drought.

Growth tests and Raman spectroscopy confirmed that DR397 is completely drought tolerant and metabolically active under drought stress (–1.25 MPa), and notably, the strain’s drought tolerance is comparatively higher than other drought-tolerant PGPR strains reported in previous studies (21, 22). Genomic analysis revealed the versatile PGP potential in contributing to IAA production, ACC deaminase activity, compatible solute accumulation and biosynthesis, ion uptake, and EPS. ACC deaminase activity and IAA production are two key traits that reduce endogenous levels of the stress hormone ethylene, thereby stimulating multiple physiological processes, such as root elongation, cell division, and enlargement under drought stress (15). The accumulation or biosynthesis of compatible solutes is one of the most efficient adaptive mechanisms in plants to maintain high turgor pressure, reduce cell osmotic potential, maintain equilibrium across the membranes, and stabilize proteins during drought (7, 10). A wide array of multiple compatible solutes, such as choline, glycine-betaine, proline, as well as glutamate uptake and the synthesis of gene clusters in DR397 allow it to act as synergistic bacteria for the improvement of plant drought tolerance.

RNA-seq analysis revealed that drought activated certain metabolic pathways in DR397. There were remarkable variations in gene expression under treatment with PEG and root exudates, whereas only subtle changes in DEGs were observed in the absence of PEG. There was an increased number of upregulated genes when treated with PEG and root exudates, which induced the biosynthesis of compatible solutes, inorganic ion transport, and defense mechanisms. We found a significant number of genes related to biosynthesis of choline, glutamine synthase, glyceryl transferase, acyltransferase, transketolase, and thiamine phosphate synthesis, which are involved in the drought tolerance of plants and plants under combined PEG and root exudate treatment (7, 23). In particular, the core mechanism of *P. fluorescence* DR397 is the production of compatible solutes after direct stimulation of plant growth under drought (Fig. 4b). Glycine betaine can stimulate symbiotic nitrogen fixation, slow down the reduction of leaf water potential, retard wilting signs, and increase the capacity to recover after stress exposure under osmotic-stressed condition (24). Choline is the precursor used for glycine-betaine synthesis and accumulation, which plays a critical role in osmoregulation (25). Enhanced accumulation of glycine-betaine and choline can improve plant growth and crop yield under drought by recruiting various physiological mechanisms such as osmotic adjustment, cellular protein stabilization, photosynthetic apparatus protection, and reduction of radical oxygen species (26). It is consisted with the previous studies that inoculation of Bacillus subtilis GB03 in *Arabidopsis* and of P. fluorescens YX2, and Raoultella planticola YL2 in maize remarkably increases choline and glycine-betaine in maize plants, resulting in improved leaf relative water content (7, 25). Based on this evidence, we speculate that the high expression of the gene clusters of glycine-betaine and choline in plant symbiosis under drought suggests the critical role of plant-bacterial interactions in alleviating drought stress in plants.

EPS biosynthesis and biofilm formation are essential for bacterial defense mechanisms and accelerating plant-bacteria interaction by root colonization under drought (6, 12). DR397 upregulates genes that degrade complex organic compounds such as alginate, benzoate, and arabinose, which promote biofilm formation and root colonization under drought stress (6, 20). Drastic upregulation of expression has been found in genes encoding the following the bifunctional enzyme UDP-glucose 4-epimerase, UDP-glucose/GDP-mannose dehydrogenase, and alginate O-acetyltransferase as key enzymes of biofilm formation (27). Lu et al. showed that the EPS-encoding gene clusters plays a vital role in the colonization of roots by Bacillus amyloliquefaciens FZB42 and induces systemic drought tolerance in Arabidopsis thaliana (28). Wang et al. reported that robust biofilm-forming mutants of B. amyloliquefaciens were positively correlated with root colonization and resistance to drought stress in tomato (20). It can be inferred that strain DR397 regulates the synthesis of EPS for inducing root colonization, and in turn, enhances the host plant’s tolerance to drought stress.

Rhizobacteria require several strategies to survive in the highly competitive microecological zones of the rhizosphere under stress (13). Flagella-mediated motility initiates biofilm formation by allowing movement, leading to the contact of bacterial cells with the surface and the subsequent secretion of the biofilm matrix components. The bacterial secretion system plays a dual role in out-competing other strains during infection of the host plant and translocating effector proteins; efflux pumps allow for the release of nutrients and secondary metabolites directly into plant cells (29). The significant upregulation of expression of gene clusters associated with flagella biosynthesis, chemotaxis, and the translocation of type VI and IV secretion systems manifested the survival strategy of strain DR397 to compete for the same ecological niche in the rhizosphere under drought. Considering all the above-mentioned genomic and transcriptomic features, we can conclude that the DR397 in plant-bacterial interactions is not an intrinsic trait of the strains; instead, it is specifically activated under drought stress.

In plant cultivation experiments, DR397 selectively promoted plant growth under drought conditions, whereas the growth improvement was not drastic under well-watered conditions. These results are cross-validation results of our hypothesis with transcriptomic profiles, that drought cause DR397 participating in plant-microbial interaction to alleviate drought stress in plant. Malik et al. proposed a revised life-history theory for microbes, named “Y-A-S,” along with two main axes of environmental variation: resources and abiotic stress (30). This theory helps to explain how drought induces the evolution of plant-bacterial interactions. Under drought stress, which imposes osmotic stress combined with limited nutrient mobility, microbes might survive in the rhizosphere using the S-A strategy. There is a preferential investment in stress tolerance mechanisms (S) to maintain cellular integrity, osmotic balance, and cellular resource acquisition (A), especially when there are scarce and complex resources (23, 30). The microbial release of compatible solutes through active secretion systems in the rhizosphere or intracellular and extracellular plant pools builds plant defenses against drought and contributes to plant growth and development under drought stress (6). Breakthrough stress survival strategies of microbes can launch unanticipated new interactions with plants, thereby building strong plant defense systems against drought. Bacterial consortia with multiple distinct drought response pathways can synergistically alleviate drought stress (6). In this context, the versatile and responsive drought-tolerant DR397 is a promising bio-inoculum to improve plant drought tolerance.

The present study presents a mechanistic approach for an in-depth exploration of microbial genetic and biochemical attributes in plant-microbe interactions under drought. Functional complementation of P. fluorescens DR397 has remarkable impacts on plant development and drought stress tolerance. Exploiting stress-responsive soil microbes and their unique stress adaptive strategies to evoke systemic tolerance that improves the metabolic capability of plants to fight abiotic stresses will lead to concerted future research on rhizosphere engineering. This study adds attributes to future research perspectives on the development of beneficial microbial consortia as an alternative sustainable strategy to improve agricultural production in extreme environments.

## MATERIALS AND METHODS

### Isolation of the strain.

Soybean plant samples were collected in June 2018 from a temporary and periodic drought-exposed agricultural land (36°58'12.0″“N 128°09'00.0”″E) in Gangwon-do province, South Korea. The plants were carefully uprooted from the soil, immediately placed in a sterile plastic bag, transported to the laboratory, and then stored at 4°C before further use. The bulk soil was removed by gently shaking the plants, and the roots were carefully washed with sterilized distilled water to collect the rhizosphere soil (5). The soil suspension was thoroughly mixed for 20 min, followed by centrifugation at 8,000 rpm for 10 min (31). To isolate root-associated bacteria, 200 μL supernatant was used to prepare decimal dilutions and the resulting aliquots (20 μL) were spread on tryptic soy agar (BD Difco, Sigma-Aldrich, USA). For preliminary study, the isolates were identified and stored in glycerol stocks at −80°C until further analysis.

### Evaluation of drought tolerance of the strain.

PEG withdraws water from the cell and cell wall, thus mimicking dry soil more closely by limiting metabolic interferences (Mahmood et al., 2012). PEG-based *in vitro* screening is the widely used suitable method to effectively screen drought tolerant crops and bacterial strains (32, 33). The drought tolerance of the isolates was evaluated by adding 25% PEG (PEG 6000; Sigma-Aldrich, MO, USA) to tryptic soy broth (TSB) (BD Difco, Sigma-Aldrich, USA) to induce an osmotic potential of −1.25 Mega Pascal (MPa) (34). The isolates were cultured in TSB medium and shaken at 120 rpm at 28 ± 2°C for 24 h. Bacterial culture (BC) of 1 × 10^7^ CFU/mL were estimated using optical density (OD_600_). These were then used as the initial inoculum and added to TSB medium supplemented with 25% PEG 6000 at a ratio of 1:10 (BC:M) (35). The OD_600_ values representing drought tolerance were determined according to previous studies: completely sensitive OD_600_ < 0.3, sensitive OD_600_ = (0.3 to 0.39), tolerant OD_600_ = (0.4 to 0.5), and completely tolerant OD_600_ > 0.5 (21).

### Raman spectroscopy.

The metabolic activity of the strain under drought (−1.25 MPa) was investigated via Raman spectroscopy using heavy water (D_2_O). D_2_O labeling and Raman measurement of strain DR397 was performed according to previously described methods using 40% D_2_O water (36).

### Biochemical characterization. (i) Quantification of IAA production.

The isolate was cultured in 5 mL (10^4^ CFU/mL) of yeast extract mannitol (BD Difco, Sigma-Aldrich, USA) liquid medium supplemented with 0.1% tryptophan. The cultures were incubated for 5 days in a shaker incubator at 120 rpm. The bacterial cultures (1 mL) were centrifuged at 10,000 rpm for 10 min. The supernatant was collected and treated with 2 mL of Salkowski reagent (2 mL 0.5 M FeCl_3_, 49 mL 70% perchloric acid, and 49 mL water). After 25 min, the absorbance of the solution was measured at 530 nm using a TECAN Spark 10 M spectrophotometer (Männedorf, Zurich, Switzerland).

**(ii) Quantitative estimation of phosphate solubilization.** Bacterial cultures (10^4^ CFU/mL) were centrifuged for 20 min at 13,000 rpm, 4°C; then, the cell pellets were collected and incubated in PSB medium at 28°C and 120 rpm. Quantitative estimation of phosphorus was performed colorimetrically in triplicate using the vanado-molybdo-phosphoric acid method (37).

**(iii) Siderophore production.** The isolate was grown on CAS medium (BD Difco, Sigma-Aldrich, USA) for 24 h to 48 h at 28°C. Siderophore production was tested qualitatively by the CAS plate assay through the detection of a yellow-orange halo around the colonies on blue CAS agar plates (38).

All experiments for biochemical characterization were performed in triplicate.

### Genomic DNA isolation and genome sequencing.

Genomic DNA was extracted and purified using the FastDNA SPIN Kit (MP Biomedicals, Solon, OH, USA), following the manufacturer’s protocol. Purified genomic DNA was sequenced at the Macrogen facility (Seoul, South Korea). The size, purity, and concentration of the samples were determined using an Agilent Technologies 2100 Bioanalyzer (Agilent Technologies Inc., Miami, FL, USA) to confirm the quality of DNA. Genome sequencing was performed using PacBio RSII (Pacific Biosciences Inc., Menlo Park, CA, USA) and Illumina HiSeq 2500 (Illumina, San Diego, CA, USA). The sequence reads were assembled using the *de novo* assembler HGAP3, and the contigs were polished using Pilon (v1.21). Genome completeness and contamination was estimated using CheckM version 1.0.0. The whole-genome sequence of strain DR397 has been deposited in the National Center for Biotechnology Information (NCBI) GenBank under the accession number CP048408 (39).

Strain DR397 was taxonomically identified by extracting the 16S rRNA sequence from whole-genome sequencing (40). The BioEdit program was used to align the sequences, and a phylogenetic tree was constructed by the neighbor-joining method using the Molecular Evolutionary Genetics Analysis (MEGA) software version 7.0 (41).

### Genome analysis and annotation.

Gene prediction and genome annotation were performed using the NCBI prokaryotic genome annotation pipeline with the best-placed reference protein set GeneMarkS 2 (v4.7) (42). Searches for related proteins were performed using the BLASTp program of NCBI with cut-off ≥98% amino acid identity and E-value < 1e-50. The complete genome sequence was submitted to the integrated microbial genome database of the Joint Genome Institute. Gene functions were further analyzed and classified into COGs (43). The ANI score between the genomic sequence of strain DR397 and other plant growth-promoting P. fluorescens strains were calculated using EZBiocloud online ANI Calculator, which was within threshold range (95% to 96%) for species (44). *In silico* analysis using antiSMASH v.5.0 revealed the presence of stress-related genes, genes involved in the production of secondary metabolites and EPS (45).

### Sample preparation for differential gene expression analysis.

Strain DR397 were grown in TSB medium in total volume of 1L by shaking at 120 rpm at 28 ± 2°C. Cells were harvested, washed three times with deionized water, inoculated into fresh M9 minimal medium (46), and grown overnight. Axenic culture (1 × 10^7^ CFU/mL) of strain DR397 were incubated for 24 h with three treatments: (i) PEG, (ii) root exudates, and (iii) PEG with root exudates in two replicates per treatment. Root exudates were prepared in a sterile manner according to Jin et al. (47). PEG treatments were conducted using 25% PEG 6000. These three treatments represent the effects of three conditions: (i) PEG, the effects of drought stress; (ii) root exudates, the effects of plant symbiosis; and (iii) PEG with root exudates, the combined effect of drought stress and plant symbiosis. Cells cultured in M9 medium without any treatment were used as controls. Physiological changes under these treatments have been measured by growth curve and Raman spectra.

### Extraction of total RNA and RNASeq analysis.

Total RNA was isolated using the RNeasy Protect Bacteria minikit (Qiagen, Hilden, Germany). The concentration and purity of RNA were determined using a NanoDrop 2000 (Thermo Fisher Scientific, Wilmington, USA). The total RNA integrity was measured using an Agilent Technologies 2100 Bioanalyzer (Agilent Technologies Inc., USA). Triplicates of RNA samples with an RNA integrity number ≥ 8 were used for sequencing by Macrogen (Seoul, South Korea). Libraries were generated using the Ribo-Zero rRNA Removal Kit (Bacteria) (Epicenter, USA) and TruSeq Standard Total RNA (NEB Microbe). The 6000 System (Illumina, San Diego, CA, USA) was used according to the user guide (Document #1000000019358 v02). Data processing was followed by Kafantaris et al. (48). DEG analyses of the PEG, root exudates, and PEG with root exudate-related bacteria, compared with the control, were performed on a comparison pair (treatment versus control) using reads per kilobase million (RPKM). Differential gene expression analysis was performed using the edgeR package to identify genes with |FC| ≥2 and a *P*-value < 0.05. Annotation and gene ontology (GO) classification for possible functional assignment of the genes was carried out based on homology with protein families of known functions after comparing their sequences to the nonredundant protein database BLASTx program with the cut-off ≥98% amino acid identity and E-value < 1e–50. *P* < 0.05 was defined as the threshold for significant enrichment of GO analyses.

### Experimental design for plant cultivation.

The capacity of DR397 to alleviate drought in two plant phenotypes (P. sativum and P. vulgaris) was evaluated via a plant cultivation test using direct inoculation. We injected 2 mL bacterial culture (1*10^^9^ CFU/mL) into each unit of plug tray containing 15 g of sterilized commercial soil (Tobirang, Baekkwang Fertility, South Korea) (49) with the properties of pH 6.8, moisture 17.2%, soil texture (clay 10.8%, silt 6.6%, and sand 82.6%), total nitrogen 3.4 ± 0.2 mg/L, total phosphate 1.4 ± 0.1 mg/L, total organic carbon 46.7 mg/L, and dissolved organic carbon 37.8 mg/L. The bacterial culture was properly mixed with soil, and one pregerminated seed was sown per unit of the plug tray. Plug trays were transferred to a growth chamber and incubated under the following conditions: 14 h/10 h light/dark cycle, 30/14°C, 50% relative humidity, and light intensity 1250/0 lx (50). Drought is the inadequate water availability to plants, which is basically generated in lab studies by pot-drying through withholding watering (51). In a preliminary study we found that the water contents of the soil without water supply for seven days was reduced to less than 50% of the water contents of the soil that supplied enough water in this growth chamber condition, which significantly lowered the plant growth (shoot length, root length, fresh weight, and dry weight) in P. sativum and P. vulgaris. Based on the preliminary data, we decided to keep plants fully watered for the first 10 days, once every alternate day, and drought stress was induced by withholding watering for next 7 days. After 7 days of drought stress, the plants were watered for 7 days and then harvested. Plants go through 7 days of drought condition considered as drought treatment, while plants grown with well-watered condition were considered as nondrought treatment. Again, plants without inoculation of the strain DR397 were used as controls for both drought and nondrought condition, respectively. At the end of the experiment, the soil was removed from the plant roots by carefully washing under low-running tap water, and the shoots and roots were separated. Shoot length, root length, and fresh and dry root weight were measured. Dry root weight was determined after drying for 24 h at 80°C. The experiment was repeated twice with 10 replicates for each treatment. Increase rate of all growth variable was calculated by comparing the growth with bacterial treatment to control under both drought and nondrought treatment.

### Statistical analysis.

Statistical analyses were performed using the R software (version 3.4.0) (52). All data from the pot test were evaluated for normality using the Shapiro-Wilk normality test. Differences between different treatments and the control group were analyzed using a two-sample *t* test. All statistical tests performed in this study were considered statistically significant at *P* ≤ 0.05.

### Data availability.

The strain was deposited at the Korean Culture Center of Microorganisms under the accession number KCCM 12710P. The whole-genome sequence of strain DR397 has been deposited in the NCBI GenBank under the accession number CP048408 (39). All sequences from RNAseq data were deposited in the Sequence Read Archive of the NCBI under the accession number (Bio project PRJNA819462; Bio sample SAMN26933189, SAMN26933190, SAMN26933191, SAMN26933192).
