# Supplementary material for: Transcriptional Response and Plant Growth Promoting Activity of Pseudomonas fluorescens DR397 under Drought Stress Conditions
Source: Microbiol Spectr. 2022 Jul 12;10(4):e00979-22. doi: 10.1128/spectrum.00979-22 (PMC9430913; doi:10.1128/spectrum.00979-22)
Supplement: Supplemental file 1 — Supplemental material. Download spectrum.00979-22-s0001.pdf, PDF file, 1.3 MB [file spectrum.00979-22-s0001.pdf]

Transcriptional response and plant growth promoting activity of *Pseudomonas fluorescens* DR397 under drought stress conditions

Nishu Susmita Das, Jee Hyun No, Tae Kwon Lee

Department of Environmental Engineering, Yonsei University, Wonju 26493, Republic of Korea

*Corresponding author

Associate Professor, Department of Environmental Engineering, Yonsei University, Wonju 26493, Republic of Korea.

E-mail address: tklee@yonsei.ac.kr (T.K. Lee)

Phone: +82-33-760-2446

Fax: +82-33-760-5524

Supplementary material

Supplementary table

Table S1 Genomic features of *P. fluorescence* DR397

**Features**	
Closest genus	Pseudomonas
Closest strain (ANI)	Pseudomonas sp. Pf0-1 (94.5%)
No of Chromosome	1
No of Plasmid	-
No of Reads	153,858
No of Contigs	1
Genome size(bp)	6,418,441
Average coverage	156
GC contents %	60.6
rRNAs	19
tRNAs	74
ncRNAs	4
Pseudo genes	32
Protein coding gene(CDS)	5,682
Accession no.	CP048408

Table S2 Secondary metabolites and extracellular polymeric substances (EPS) produced by *P. fluorescence* DR397

	Name	Function
Secondary metabolite	NAGGN	Osmoprotectants
	Arylpolyene	Oxidative stress or ROS protectants
	Bacteriocin	Peptidic toxins,inhibit the growth of similar or closely related bacterial strain
	Terpene	Pesticide
	Lokisin	Antifungal activity
	Siderophore	High-affinity iron-chelating compounds
Extracellular polymeric substances(EPS)	Alginate	Capsular polysaccharides<br>Matrix molecule for biofilm formation by providing more structural stability. Robust biofilm integrity relates to the ability to adhere to surfaces, aggregation to neighboring biofilm cells
	Cellulose	Aggregative polysaccharides<br>Impacts biofilm formation at the meniscus and air-liquid (A-L) interface

Table S3 The Raman frequency with significantly different intensity in the spectra of DR397 cells under different treatments.

Treatments	Frequency	Assignment	Reference
PEG25%+Root exudates	853	Protein (glycogen, collagen)	(1, 2)
Root Exudates	853	Protein (glycogen, collagen)	(1, 2)
PEG25%+Root exudates	922	L-alanine	(2)
Root Exudates	1002	Phenylalanine, b-carotene	(3)
PEG25%+Root exudates	1002	Phenylalanine, b-carotene	(3)
PEG25%	1054	Nucleic acids, Protein	(4)
PEG25%+Root exudates	1054	Nucleic acids, Protein	(4)
PEG25%	1240	Thymine, cytosine, adenine	(4)
PEG25%	1242	Amide III	(3)
PEG25%	1246	Thymine, cytosine, adenine	(5)
PEG25%	1375	Thymine, adenine, guanine	(4)
Root Exudates	1441	Lipids	(6)
Root Exudates	1450	Nucleic acid, protein, lipid, carbohydrate	(1)
Root Exudates	1482-1487	Nucleic acids	(7)
PEG25%+Root exudates	1573	Guanine, Adenine; Amide II	(3)
PEG25%	1573	Guanine, Adenine; Amide II	(3)
PEG25%	1593	Protein	(8)
PEG25%	1599	Phenylalanine, tyrosine	(1, 9)

Supplementary figure

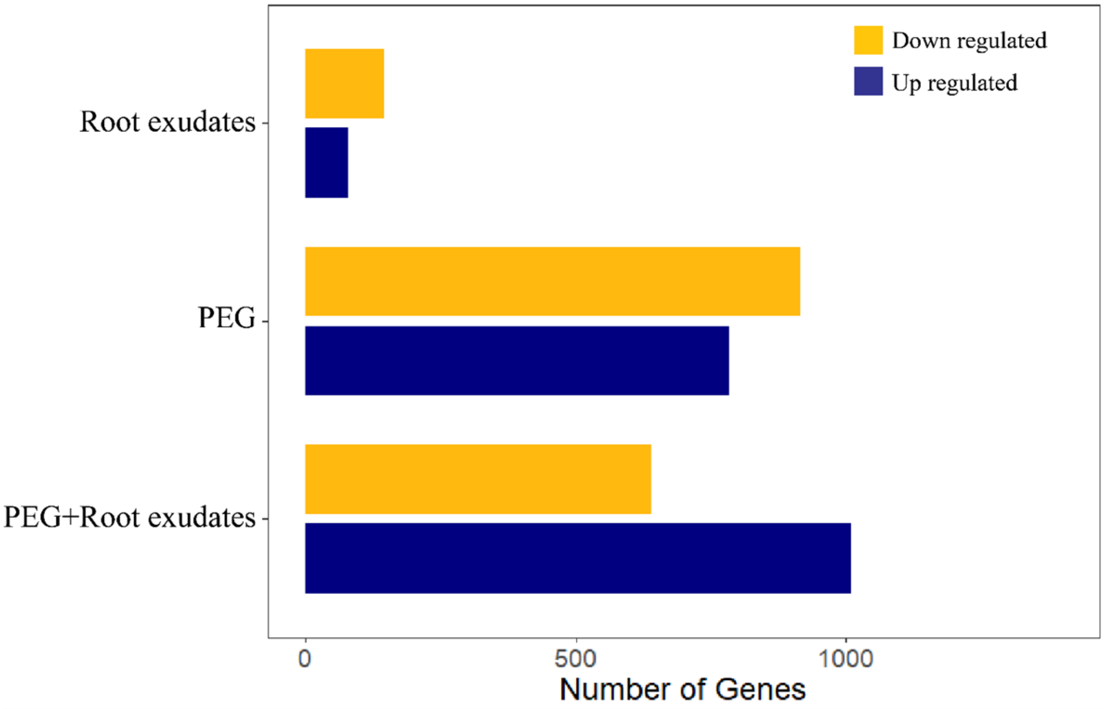

FIG S1 Transcriptomic modulation in *P. fluorescence* DR397 genes in response to drought stress and plant bacteria symbiosis. Gene expression was analyzed via RNAseq, under three treatments (Root exudates, PEG and PEG with root exudates) with two replicates of each. After filtering genes from raw data with low quality through the data preprocessing and QC process, TMM normalization was done. For statistical analysis, fold change and exact test using edge were used for each treatment. Gene expression was measured by comparing the fold change (FC) under treatment vs control. Statistically significant genes were chosen which meets the criteria (lFCl>=2 and p<0.05) in both replicates. The bars showing in this figure represents the number of significant genes expressed under each treatment; Yellow color indicates down regulated genes and blue color indicates up regulated genes. PEG, Root exudates and PEG+Root exudates represents the effect of drought, plant symbiosis and drought along with plant symbiosis respectively.

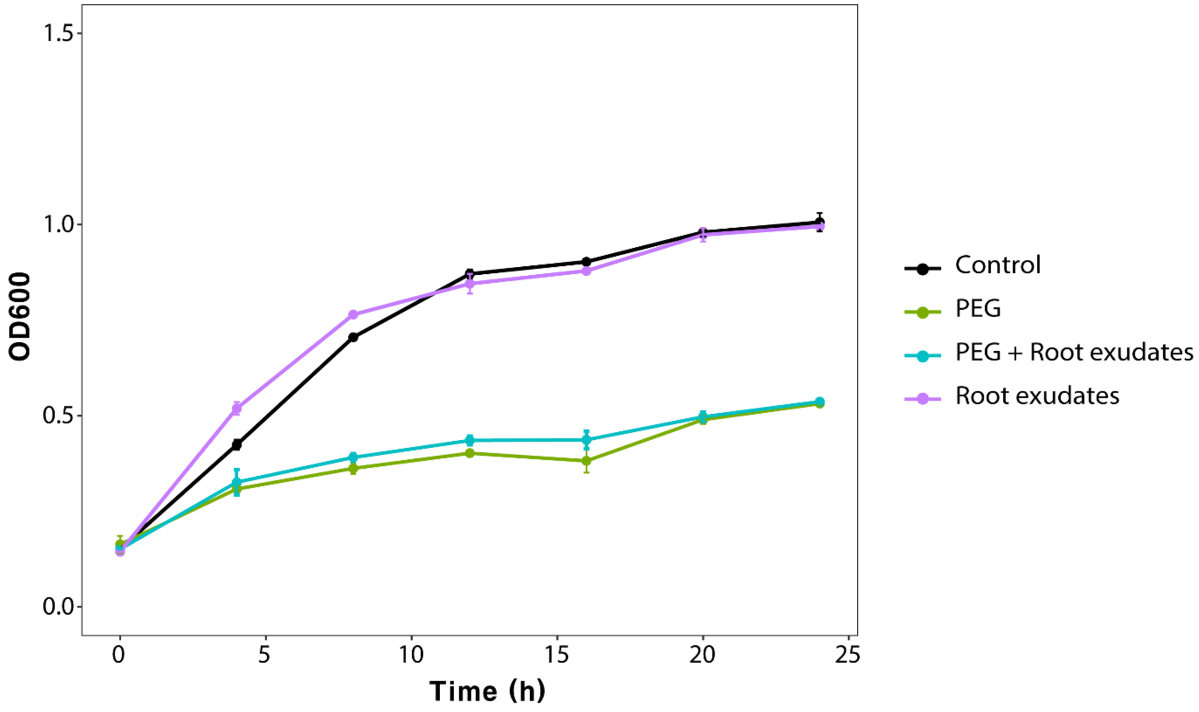

FIG S2 Growth curve of *P. fluorescence* DR397 treated with i. 25% PEG, ii. 25% PEG+Root Exudates, iii. Root Exudates till 24 h. Cells cultured in M9 medium without any treatment were used as controls.

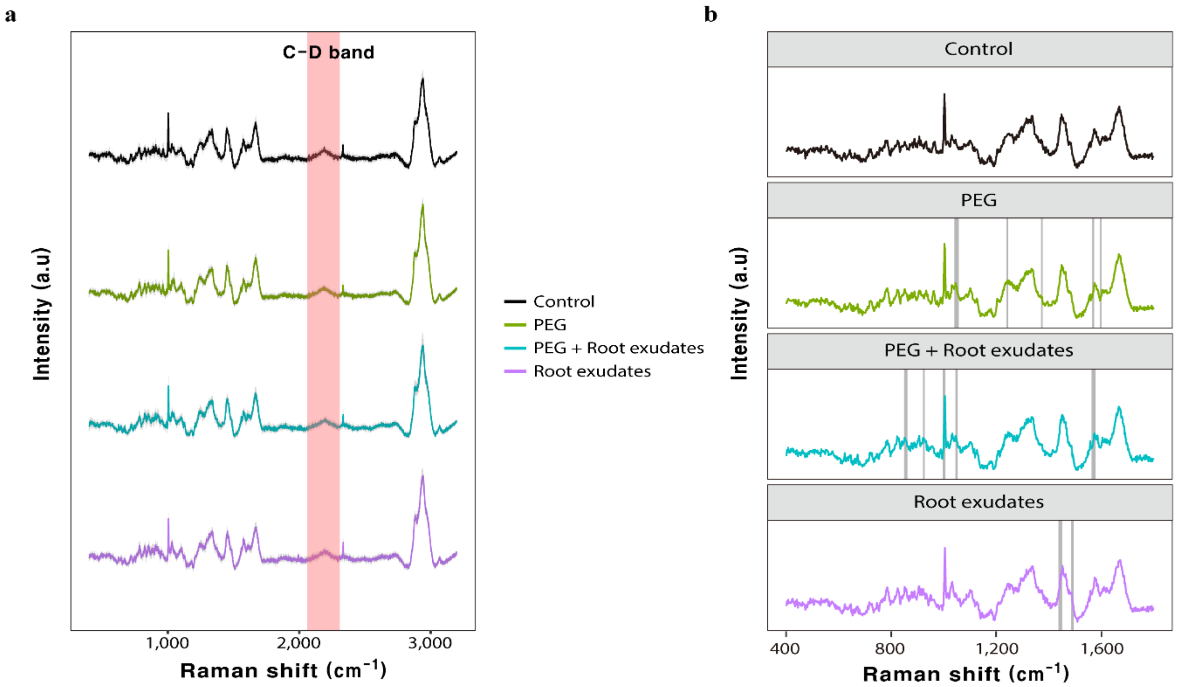

FIG S3 Single-cell Raman spectra (SCRS) of *P. fluorescence* DR397. a. Raman spectra of DR397 cell after 24 h of incubation in medium with i. 25% PEG, ii. 25% PEG+Root Exudates, iii. Root Exudates including 40% deuterium water. Cells cultured in M9 medium without any treatment were used as controls. b. Comparison of the Raman fingerprint section between different treatments and control (t test, $p < 0.01$). Grey parts are representing the difference in peaks between the treatments to control.

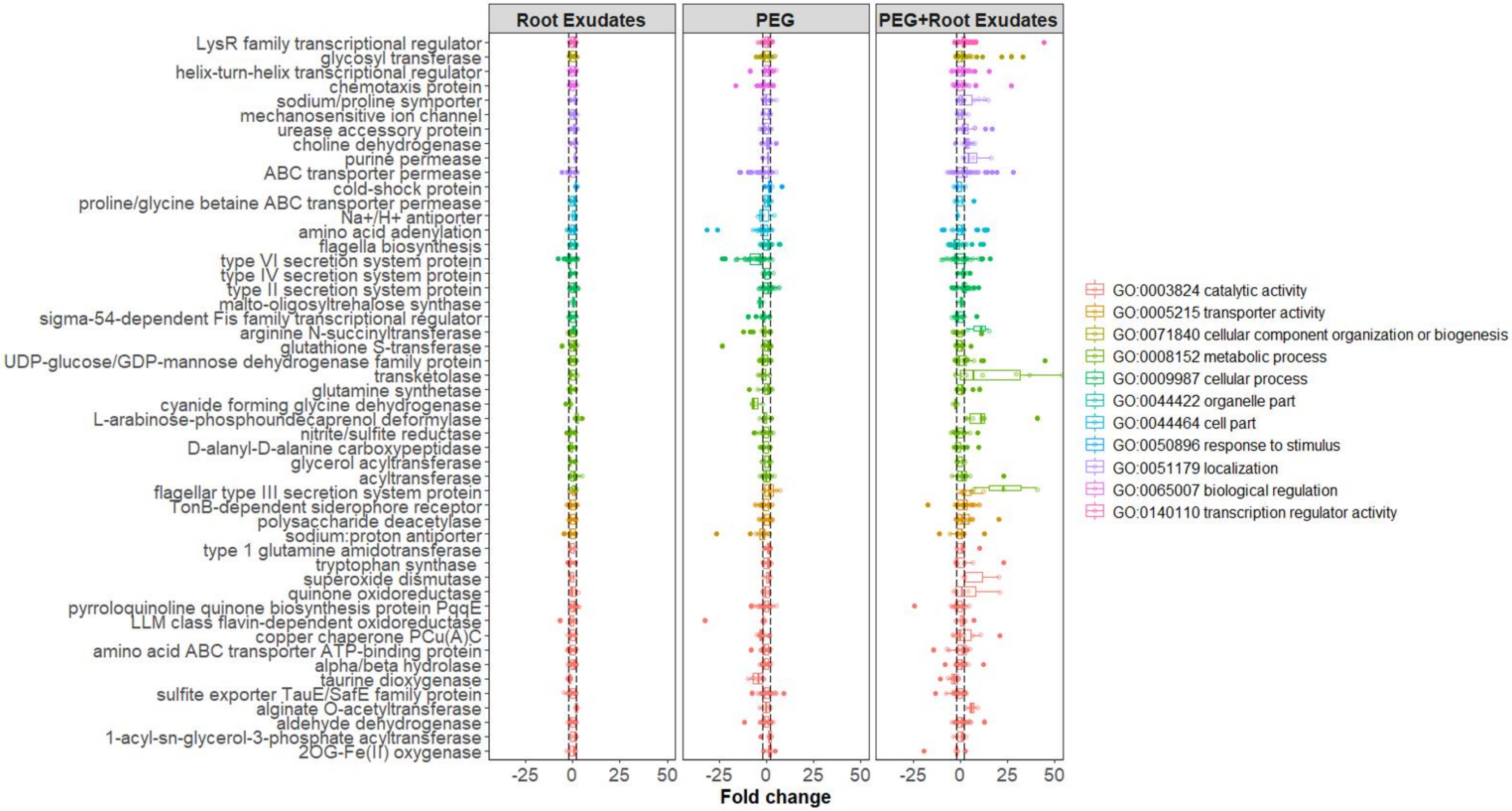

FIG S4 Gene expression analysis of highly regulated functions in *P. fluorescence* DR397. Fold changes in most up- and down-regulated genes in *P. fluorescence* DR397 under the treatment of Root exudates, PEG, and PEG+Root exudates. Fold changes with the treatment of PEG represents the effects of drought stress while PEG+Root exudates represent drought stress along with plant symbiosis on DR397 genes. DR397 incubation in media is considered as control for gene expression analysis. Both size and colors of the circle representing the FC values.

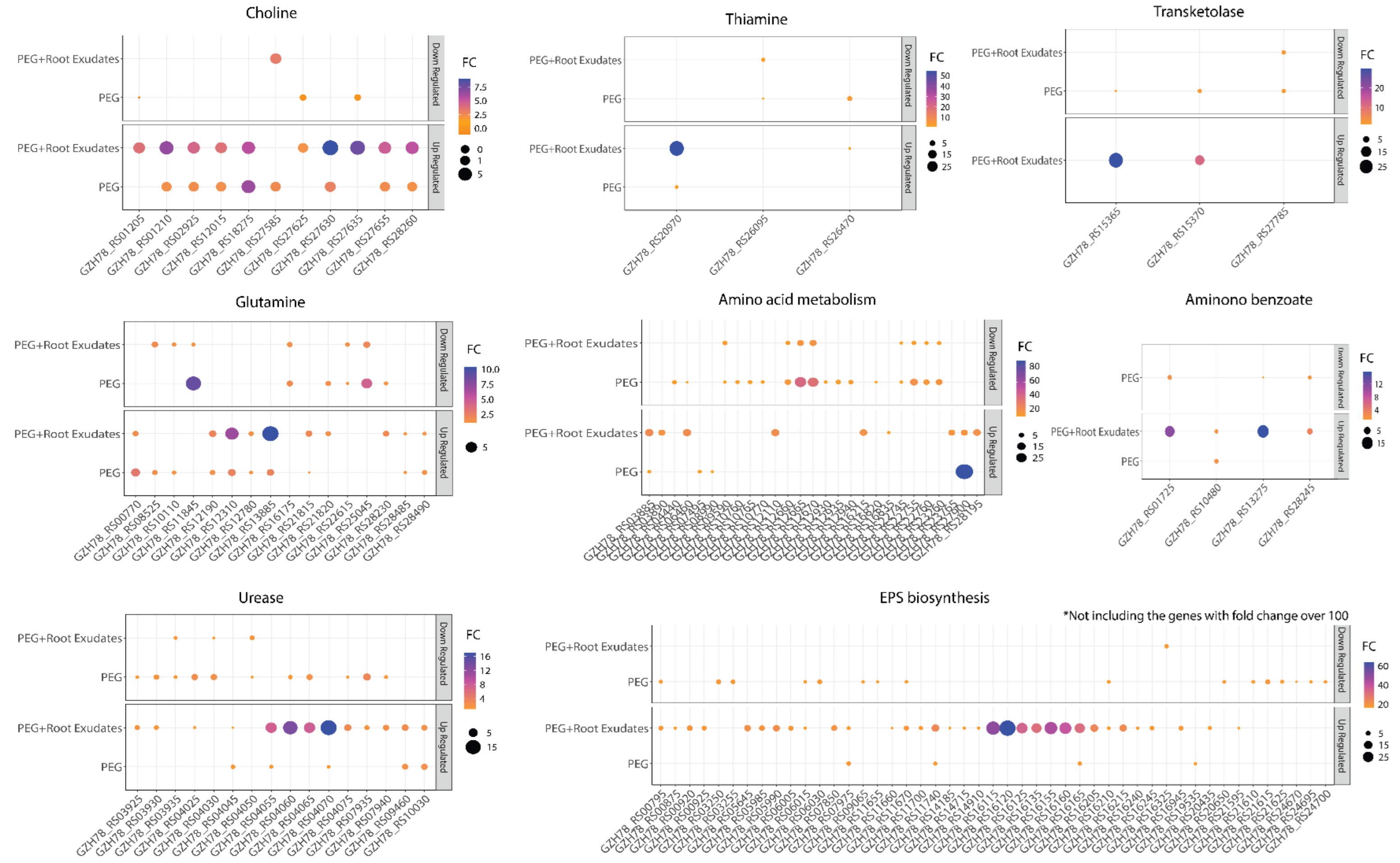

FIG S5 Fold changes of DR397 genes corporates in synthesis and metabolism of plant drought regulatory functions. Significantly regulated gene clusters belong to biosynthesis of Choline, Thiamine, Transketolase, Glutamine, Amino acid metabolism, Amino benzoate, Urease, EPS biosynthesis, in *P. fluorescence* DR397 under the treatment of PEG and PEG+Root exudates. Both size and colors of the circle representing the FC values.

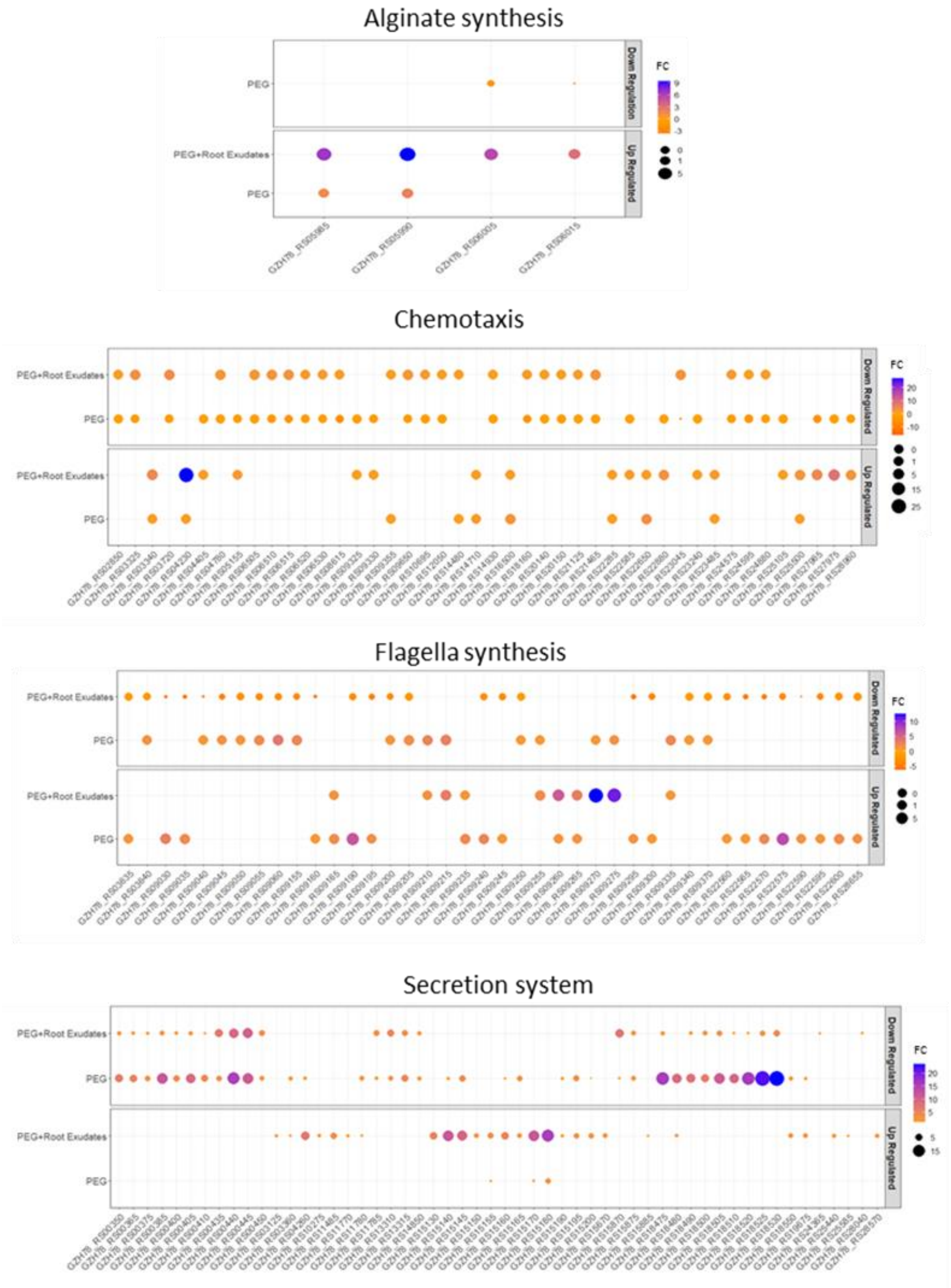

FIG S6 Expressed gene related biofilm formation and colonization. Fold changes of genes related Alginate synthesis, Chemotaxis, Flagella synthesis, Secretion system in *P. fluorescence* DR397 under the treatment of PEG and PEG+Root exudate. Both size and colors of the circle representing the FC values.

Reference mentioned in supplementary data

1. Notingher I, Hench LL. 2006. Raman microspectroscopy: a noninvasive tool for studies of individual living cells in vitro. Expert review of medical devices 3:215-234.
2. De Gelder J, De Gussem K, Vandenabeele P, Moens L. 2007. Reference database of Raman spectra of biological molecules. Journal of Raman Spectroscopy: An International Journal for Original Work in all Aspects of Raman Spectroscopy, Including Higher Order Processes, and also Brillouin and Rayleigh Scattering 38:1133-1147.
3. Strola S, Marcoux P, Schultz E, Perenon R, Simon A-C, Espagnon I, Allier C, Dinten J. Differentiating the growth phases of single bacteria using Raman spectroscopy, p 16-24. *In* (ed), SPIE,
4. Uzunbajakava N, Lenferink A, Kraan Y, Willekens B, Vrensen G, Greve J, Otto C. 2003. Nonresonant Raman imaging of protein distribution in single human cells. Biopolymers: Original Research on Biomolecules 72:1-9.
5. Huang WE, Li M, Jarvis RM, Goodacre R, Banwart SA. 2010. Shining light on the microbial world: the application of Raman microspectroscopy. Advances in applied microbiology 70:153-186.
6. van Manen H-J, Kraan YM, Roos D, Otto C. 2005. Single-cell Raman and fluorescence microscopy reveal the association of lipid bodies with phagosomes in leukocytes. Proceedings of the National Academy of Sciences 102:10159-10164.
7. Schuster KC, Urlaub E, Gapes J. 2000. Single-cell analysis of bacteria by Raman microscopy: spectral information on the chemical composition of cells and on the heterogeneity in a culture. Journal of Microbiological Methods 42:29-38.
8. Maquelin K, Kirschner C, Choo-Smith L-P, van den Braak N, Endtz HP, Naumann D, Puppels G. 2002. Identification of medically relevant microorganisms by vibrational spectroscopy. Journal of microbiological methods 51:255-271.
9. Teng L, Wang X, Wang X, Gou H, Ren L, Wang T, Wang Y, Ji Y, Huang WE, Xu J. 2016. Label-free, rapid and quantitative phenotyping of stress response in E. coli via ramanome. Scientific reports 6:1-10.
